# Development of Mid-Infrared Spectroscopy (MIR) Diagnostic Model for Udder Health Status of Dairy Cattle

**DOI:** 10.3390/ani15152242

**Published:** 2025-07-30

**Authors:** Xiaoli Ren, Chu Chu, Xiangnan Bao, Lei Yan, Xueli Bai, Haibo Lu, Changlei Liu, Zhen Zhang, Shujun Zhang

**Affiliations:** 1 Key Lab of Agricultural Animal Genetics, Breeding and Reproduction of Ministry of Education, Huazhong Agricultural University, Wuhan 430070, China; 2 Frontiers Science Center for Animal Breeding and Sustainable Production, Huazhong Agricultural University, Wuhan 430070, China; 3 Henan Provincial International Joint Laboratory for Dairy Health Farming, Henan Dairy Herd Improvement Center, Zhengzhou 450046, China; 4 Henan Seed Industry Development Center, Zhengzhou 450046, China; 5 College of Animal Science and Technology, China Agricultural University, Beijing 100193, China; 6 Henan Dairy Herd Improvement Co., Ltd., Zhengzhou 450046, China

**Keywords:** somatic cell count, differential somatic cell count, mid-infrared spectroscopy, dairy cattle

## Abstract

The somatic cell count (SCC) and differential somatic cell count (DSCC) are proxies for the udder health of dairy cattle, regarded as the criterion of mastitis identification with healthy, suspicious mastitis, mastitis, and chronic/persistent mastitis. However, SCC and DSCC are tested using flow cytometry, which is expensive and time-consuming, particularly for DSCC analysis. Mid-infrared spectroscopy (MIR) enables qualitative and quantitative analysis of milk constituents with great advantages, being cheap, non-destructive, fast, and high-throughput. The objective of this study is to develop a dairy cattle udder health status diagnostic model of MIR. Data on milk composition, SCC, DSCC, and MIR from 2288 milk samples collected in dairy farms were analyzed using the CombiFoss 7 DC instrument (FOSS, Hilleroed, Denmark). Three MIR spectral preprocessing methods, six modeling algorithms, and three different sets of MIR spectral data were employed in various combinations to develop several diagnostic models for mastitis of dairy cattle. The MIR diagnostic model of effectively identifying the healthy and mastitis cattle was developed using a spectral preprocessing method of difference (DIFF), a modeling algorithm of Random Forest (RF), and 1060 wavenumbers, abbreviated as “DIFF-RF-1060 wavenumbers”, and the AUC reached 1.00 in the training set and 0.80 in the test set. The other MIR diagnostic model of effectively distinguishing mastitis and chronic/persistent mastitis cows was “DIFF-SVM-274 wavenumbers”, with an AUC of 0.87 in the training set and 0.85 in the test set. For more effective use of the model on dairy farms, it is necessary and worthwhile to gather more representative and diverse samples to improve the diagnostic precision and versatility of these models.

## Simple Summary

The objective of this study is to develop a mid-infrared spectroscopy (MIR) diagnostic model for udder health status in dairy cattle. Somatic cell count (SCC) and differential somatic cell count (DSCC), measured in milk samples, are used to classify udder health. The DIFF-RF-1060 wavenumbers model distinguished healthy cattle from those with mastitis, achieving an AUC of 0.80 in the test set. The DIFF-SVM-274 wavenumbers model further differentiated mastitis from chronic/persistent mastitis cases, with an AUC of 0.85 in the test set.

## 1. Introduction

Mastitis, caused by intramammary infection, reduces milk production, increases culling rates, and decreases the profitability of dairy farming [1,2,3]. Mastitis is categorized into clinical and subclinical forms. Economic losses attributable to clinical mastitis range from USD 12,000 to USD 76,000 per farm per month [4]. Somatic cell count (SCC) is the total number of polymorphonuclear neutrophils (PMN), macrophages (MAC), and lymphocytes (LYM) in milk. It serves as a critical indicator for assessing udder health and milk quality [5]. Additionally, SCC is an indicator for subclinical mastitis [6]. Research indicates that the SCC in milk increases when the mammary gland is infected by pathogens [3], altering the proportions of PMN, LYM, and MAC; however, SCC alone does not reflect these changes in cell proportions [7]. The differential somatic cell count (DSCC) represents the percentages of PMN and LYM within the total SCC in milk [5,8].

Somatic cell count (SCC) and differential somatic cell count (DSCC) more accurately reflect the dynamic changes in the udder health status of dairy cattle and can be used for the early detection of mastitis [9]. According to Schwarz, using SCC of 200$\;\times {\;10}^{3}$ cells/mL and DSCC of 65% as criteria [10], the udder health groups are as follows: (1) Group A: SCC ≤ 200$\;\times \;10^{3}$ cells/mL and DSCC ≤ 65%, indicating healthy cattle; (2) Group B: SCC ≤ 200 $\times \;10^{3}$ cells/mL and DSCC > 65%, indicating suspicious mastitis, posing a risk of developing the condition, requiring dairy farm managers to pay close attention to these cows’ health; (3) Group C: SCC > 200$\;\times {\;10}^{3}$ cells/mL and DSCC > 65%, indicating mastitis, necessitating treatment measures; (4) Group D: SCC > 200$\;\times {\;10}^{3}$ cells/mL and DSCC ≤ 65%, indicating chronic/persistent mastitis, where, in addition to treatment, farm managers might consider culling these cows according to their lactation stage and production performance.

Somatic cell count (SCC) and differential somatic cell count (DSCC) are primarily measured using flow cytometry. DSCC can only be measured using the CombiFoss7 DC instrument (FOSS, Hillerød, Denmark) [8], which is expensive to purchase and maintain. Mid-infrared spectroscopy (MIR) operates within the wavelength range of 2500 nm to 15,000 nm, analyzing the energy absorption of chemical bonds or functional groups of organic materials in the mid-infrared spectrum to qualitatively and quantitatively assess the composition of cattle milk. Mid-infrared spectroscopy (MIR), known for its low-cost, non-destructive, rapid, and high-throughput characteristics, is widely used in Dairy Herd Improvement (DHI) to measure components such as milk fat, protein, and lactose [11,12]. Mid-infrared spectroscopy (MIR) is also used to diagnose and monitor specific substances and health conditions in dairy cattle. These include milk fatty acids [13], milk coagulation properties [14], protein composition [15], minerals [16], ketosis [17], and lameness [18]. Somatic cell count (SCC) and differential somatic cell count (DSCC) cannot be directly measured using mid-infrared spectroscopy [19], although studies have shown a negative correlation between milk composition and both SCC [20] and DSCC [21]. Milk from cows with higher SCC value exhibits higher electrical conductivity and pH due to elevated levels of Na^+^ and Cl^−^ ions and decreased K^+^ content [3]. Furthermore, there are few studies about classification and diagnostic models for mastitis using MIR. Therefore, the objective of this study is to develop a diagnostic MIR model for cattle mastitis.

## 2. Materials and Methods

### 2.1. Dairy Herd

The study focused on Holstein dairy cattle raised on a farm in the Central China region. The dairy farm was situated in an area characterized by a warm temperate to subtropical climate, with conditions ranging from humid to semi-humid under the influence of monsoon weather patterns. Cows were fed a Total Mixed Ration (TMR) and had access to water through automatic drinkers installed in the barn, with modern management practices in place. Daily animal welfare monitoring by certified herd managers was integrated with digital record-keeping via herd management software. Nutritional formulations, reproductive strategies, and genetic selection programs followed evidence-based protocols supervised by agricultural institution specialists and non-financial technical units.

### 2.2. Sample Collection and Testing

From March 2019 to December 2021, 2288 milk samples from 1010 cattle were collected from one farm, adhering to the guidelines of the International Committee for Animal Recording (ICAR). Milk samples from lactating cows were collected monthly from milking parlors on the farm. Samples were preserved with bronopol (2-bromo-2-nitro-1,3-propanediol), ensuring stability for SCC/DSCC and compositional assays. Each sample ranged from 30 mL to 50 mL and was immediately mixed with preservatives after collection and transported to the Dairy Herd Improvement (DHI) laboratory in Zhengzhou, Henan, China, for testing with the CombiFoss 7 DC instrument (FOSS, Hilleroed, Denmark). The data collected included milk yield, milk composition, SCC, DSCC, and MIR data.

### 2.3. Data Processing and Analysis

To enhance the model’s effectiveness, the data for modeling were selected according to the following criteria: (1) days in milk between 5 d and 365 d; (2) milk fat and protein percentages between 1.50% and 9.00%. The refined modeling data included 2116 milk samples from 983 dairy cattle. First-, second-, and third-parity groups comprised 853, 654, and 609 samples, respectively.

The criterion for diagnosing mastitis combined SCC and DSCC, setting thresholds at 200$\;\times {\;10}^{3}$ cells/mL and 65% DSCC, respectively, dividing the samples into four groups [10]: Group A (healthy: 0 ≤ SCC ≤ 200$\;\times \;10^{3}$ cells/mL and DSCC ≤ 65%), Group B (suspicious mastitis: SCC ≤ 200$\;\times \;10^{3}$ cells/mL and DSCC > 65%), Group C (mastitis: SCC > 200$\;\times \;10^{3}$ cells/mL and DSCC > 65%), and Group D (chronic/persistent mastitis: SCC > 200$\;\times \;10^{3}$ cells/mL and DSCC ≤ 65%).

The impact of different groups on milk yield and composition was analyzed by a linear mixed model using the lme4 package (version 1.1.35) [22] in R software (version 4.4.0) [23]. Multiple comparisons were conducted using the lsmeans package (version 2.30.0) [24] in R software (version 4.4.0) [23]. The analytical model was as follows:

(1) $$y_{ijklm}={Par}_{i}+{Dim}_{j}+{Group}_{k}+a_{l}+e_{ijklm}$$

where $y_{ijklm}$ represents milk yield and milk composition; ${Par}_{i}$ indicates the effect of parity, with parities grouped into three categories: first parity, second parity, and third or subsequent parities; ${Dim}_{j}$ represents the effect of days in milk at the time of sample testing, grouped into three categories: 1–100 days, 101–200 days, and over 201 days; ${Group}_{k}$ denotes the grouping according to the criterion of mastitis identification, where samples were classified into groups A, B, C, and D using the criterion of mastitis identification of SCC and DSCC; $a_{l}$ represents cow random effects; $e_{ijklm}$ denotes the residuals.

### 2.4. Spectral Modeling and Evaluation

#### 2.4.1. Spectral Preprocessing and Feature Extraction

Scattering from casein micelles and random noise generated during instrument operation can interfere with spectral data, which not only contain valuable chemical information but also a significant amount of background noise and irrelevant information. To eliminate systematic errors caused by the environment, instrument, and operation during spectral collection, preprocessing of the spectra was required before modeling. This study employed three spectral preprocessing methods: Difference (DIFF), Savitzky–Golay convolution smoothing (SG), and no preprocessing (None), with only the results from the optimal spectral preprocessing method presented.

The mid-infrared spectroscopy (MIR) of milk consists of 1060 distinct wavenumbers within the range of 925 to 5008 ${cm}^{-1}$, featuring high dimensionality and some overlap between different spectral bands. Using feature extraction algorithms can significantly reduce the spectral dimensions, enhance modeling speed, and eliminate noise from spectra. This paper utilizes the full spectrum of 1060 wavenumbers, the internationally used 274 informative wavenumbers for milk MIR modeling (925 to 1584 ${cm}^{-1}$, 1719 to 1784 ${cm}^{-1}$, and 2652 to 2976 ${cm}^{-1}$, hereafter referred to as “274 informative wavenumbers”), and the EU-recommended 212 wavenumbers (964 to 1574 ${cm}^{-1}$; 1728 to 1759 ${cm}^{-1}$; 1778 to 1801 ${cm}^{-1}$; 2827 to 2996 ${cm}^{-1}$). To enhance the model’s applicability across diverse herd management systems, only MIR data were utilized for modeling, with lactation stage excluded.

#### 2.4.2. Model Establishment

Before modeling, 80% of the data was selected randomly and used as the training set (Train) to fit the models, and 20% as the test set (Test) to evaluate model performance. This study employed six modeling algorithms: Random Forest (RF) [25], K-Nearest Neighbor (KNN) [26], Linear Regression (LR), Naive Bayes Model (NBM) [27], Adaptive Boosting (Adaboost) [28], and Support Vector Machine (SVM) [29]. All machine learning algorithms were implemented using Python 3 [30], with only the results from the best-performing modeling algorithm displayed.

#### 2.4.3. Model Evaluation

The models developed in this study were categorized into Dichotomous and multiclass diagnostics. The evaluation metrics for dichotomous classification models include accuracy (ACCU), sensitivity (SENS), specificity (SPEC), positive predictive value (PPV), negative predictive value (NPV), Matthews correlation coefficient (MCC), and area under the receiver operating characteristic curve (AUC). The calculation methods for these metrics were as follows:

(2) $$SENS=\frac{TP}{TP+FN}$$

(3) $$SPEC=\frac{TN}{TN+FP}$$

(4) $$ACCU=\frac{TP+TN}{TP+TN+FP+FN}$$

(5) $$PPV=\frac{TP}{TP+FP}$$

(6) $$NPV=\frac{TN}{TN+FN}$$

(7) $$MCC=\frac{TP\times TN-FP\times FN}{\sqrt{\left(TP+FP\right)\left(TP+FN\right)(TN+FP)(TN+FN)}}$$

where true positives (*TP*) represent the number of correct positive predictions; false positives (*FP*) represent the number of incorrect positive predictions; true negatives (*TN*) represent the number of correct negative predictions; false negatives (*FN*) represent the number of incorrect negative predictions.

## 3. Results

### 3.1. The Differences in Milk Yield and Composition Among the Different Mastitis Cattle

**Figure 1:**
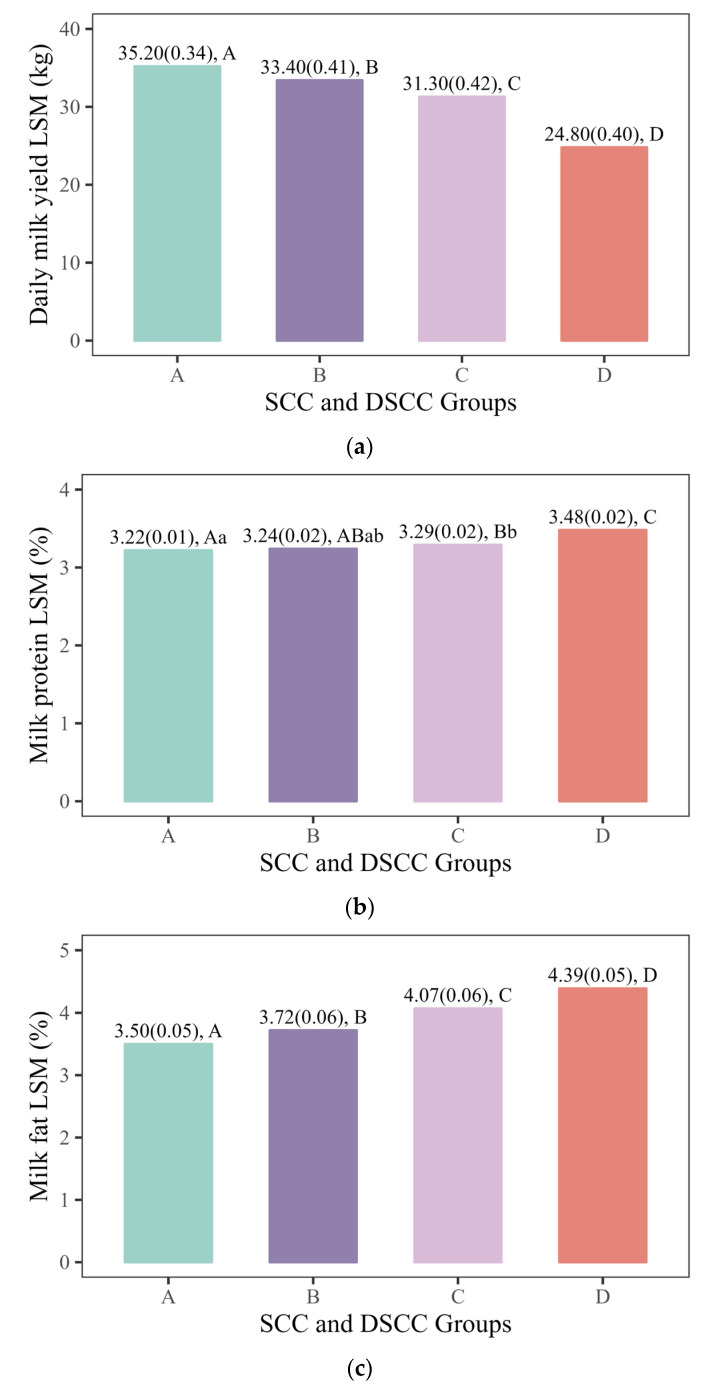

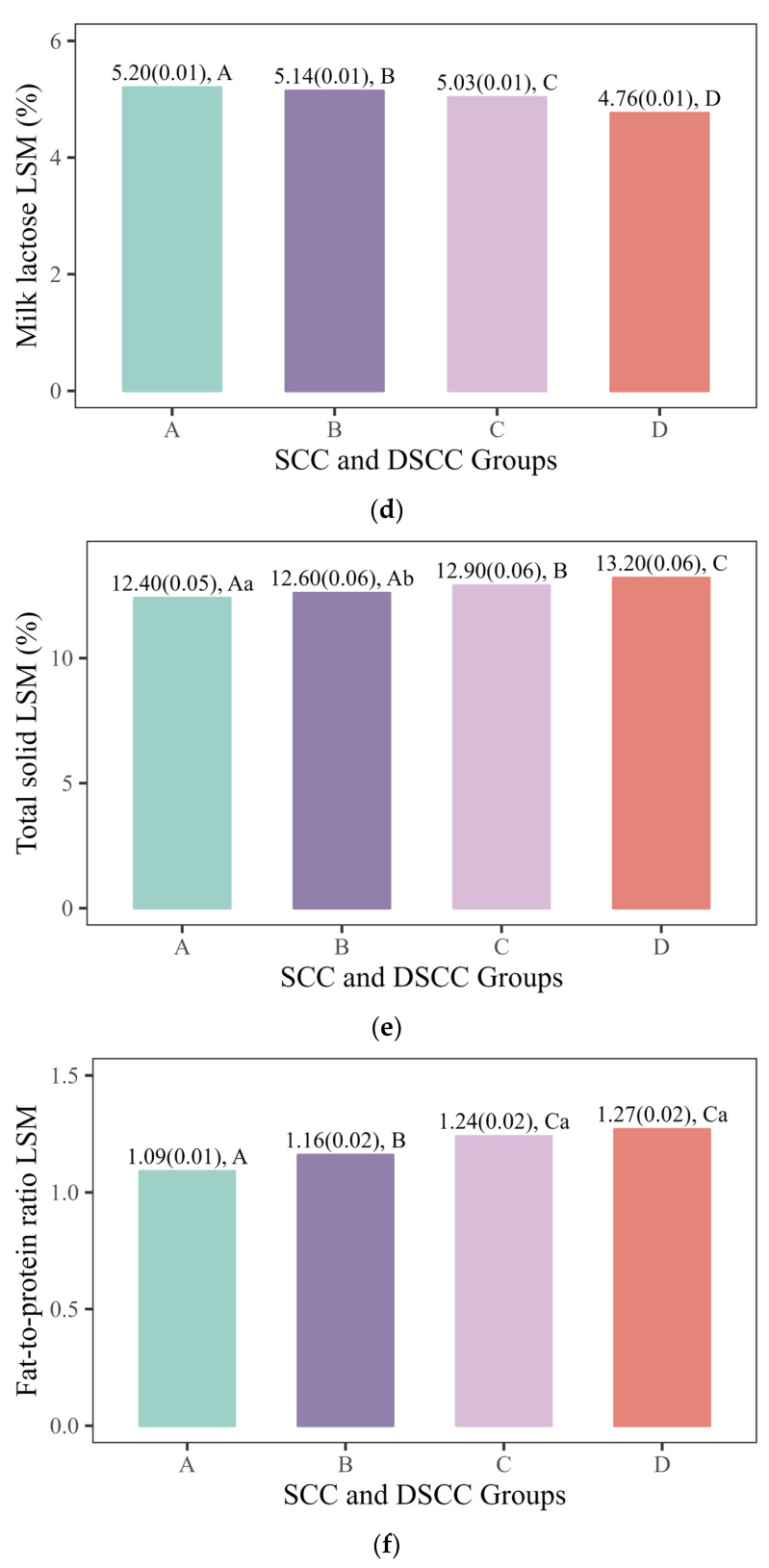
Least squares mean of milk yield and milk composition in different groups using the criterion of mastitis identification of SCC and DSCC. The data consist of the least squares means and corresponding standard errors. Letters indicate whether there were significant differences between different groups. Same letters, different lowercase letters, and different uppercase letters mean no significant difference (*p* > 0.05), significant difference (*p* < 0.05), and highly significant difference (*p* < 0.01), respectively. (**a**) Daily milk yield, (**b**) milk protein, (**c**) milk fat, (**d**) milk lactose, (**e**) total solid, (**f**) fat-to-protein ratio.

There were significant differences in milk yield and composition among the different SCC and DSCC groups (Figure 1). Differences in milk yield, milk fat percentage, and lactose percentage were highly significant (*p* < 0.01) across Groups A, B, C, and D. Protein percentage shows no significant difference (*p* > 0.05) between Groups A and B, but was significantly or highly significantly different (*p* < 0.05 or *p* < 0.01) among the other groups. Total solids exhibit significant differences (*p* < 0.05) between Groups A and B, and highly significant differences (*p* < 0.01) among the other groups. The fat-to-protein ratio shows no significant difference (*p* > 0.05) between Groups C and D, but was highly significant (*p* < 0.01) among the other groups.

### 3.2. MIR Diagnostic Model for Healthy and Mastitis Cattle

**Figure 2:**
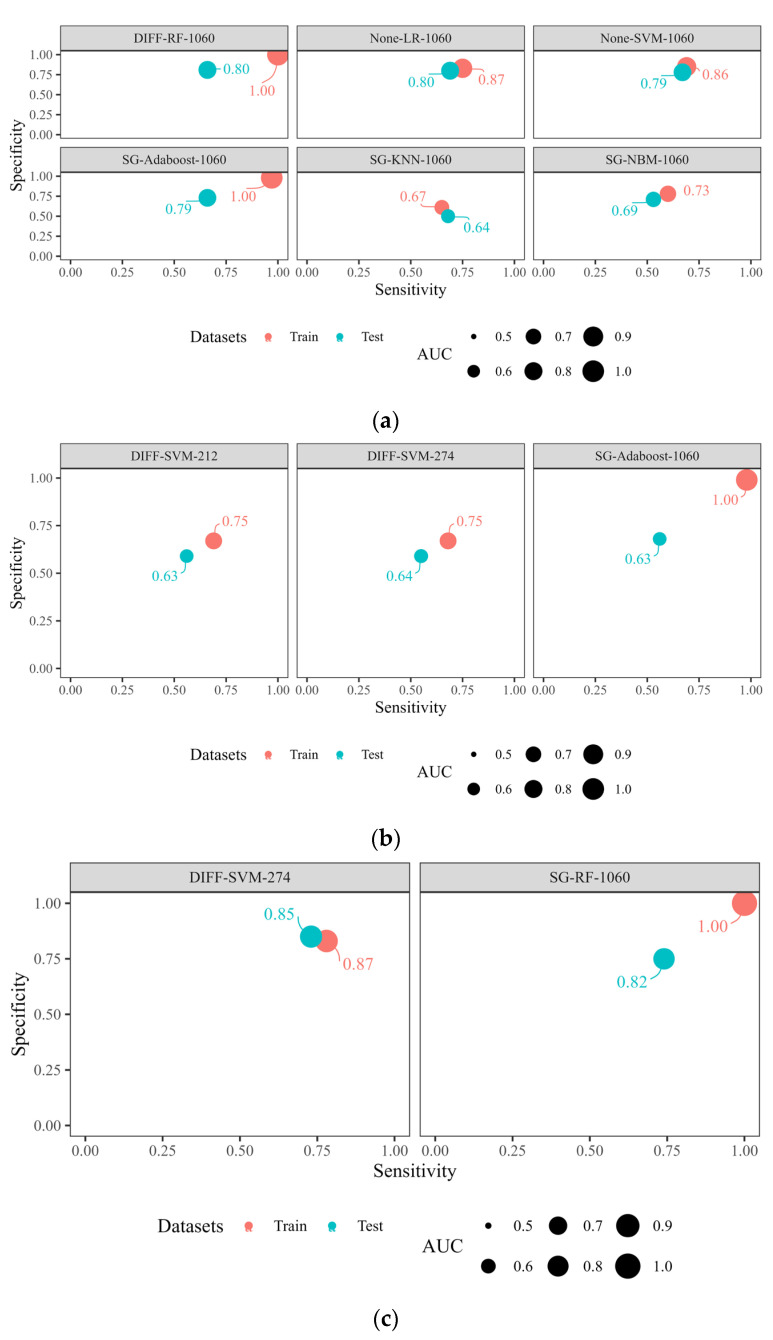
Specificity, sensitivity, and AUC of two-class MIR detection model of dairy cattle udder health using the criterion of mastitis identification of SCC and DSCC. (**a**) MIR model for healthy and mastitis. (**b**) MIR model for healthy and suspicious mastitis. (**c**) MIR model for mastitis and chronic/persistent mastitis. Modeling algorithms: KNN represents K-nearest neighbor, NBM represents Naive Bayes model, RF represents Random Forest, SVM represents support vector machine, LR represents linear regression, Adaboost represents adaptive boosting. Spectral preprocessing methods: SG represents Savitzky–Golay convolution smoothing, DIFF represents Difference, None represents no preprocessing for MIR data. Model evaluation metrics: ACCU represents accuracy, SENS represents sensitivity, SPEC represents specificity, PPV represents positive predictive value, NPV represents negative predictive value, MCC represents Matthews correlation coefficient, AUC represents area under receiver operating characteristic curve. Only MIR data were utilized for modeling, with lactation stage excluded.

The milk samples were divided into healthy (Group A) and mastitis cows (Group BCD), with each group consisting of 600 samples. Table 1 and Figure 2 summarize the performance of the dichotomous models based on MIR data, excluding lactation stage, with SCC and DSCC as predictors. The modeling approach involved three spectral preprocessing methods (DIFF, SG, and None) and six spectral modeling algorithms (RF, KNN, LR, NBM, Adaboost, and SVM) across three different spectral wavenumbers (the full spectrum of 1060 wavenumbers, 274 feature wavenumbers, and the EU-recommended 212 wavenumbers). The models were compared and analyzed to identify the most effective one. Six MIR diagnostic models have been established for healthy and mastitis cows. Compared with other models, the model “DIFF-RF-1060 wavenumbers”, using the spectral preprocessing method with difference (DIFF), a modeling algorithm with random Forest (RF), and 1060 wavenumbers, demonstrated high predictive performance, with AUCs of 1.00 in the training set and 0.80 in the test set.

### 3.3. MIR Diagnostic Model for Healthy and Suspicious Mastitis Cattle

The MIR modeling approach, excluding lactation stage, for healthy (Group A) and suspicious mastitis (Group B) cattle was similar to that for healthy and mastitis cattle. The number of healthy and suspicious mastitis cattle was 400 in each group. From Table 1 and Figure 2, the “SG-Adaboost-1060 wavenumbers” model, using the spectral preprocessing method Savitzky–Golsy convolution smoothing (SG), a modeling algorithm with adaptive boosting (Adaboost), and 1060 wavenumbers, the AUCs of the model of distinguishing healthy from suspicious mastitis cattle showed 1.00 in the training set and 0.63 in the test set. However, this model demonstrated poor performance in distinguishing between healthy and suspicious mastitis cattle, with an AUC in the test set lower than 0.70.

### 3.4. MIR Diagnostic Model for Mastitis and Chronic/Persistent Mastitis Cattle

After dividing the 800 cattle into mastitis (Group C) and chronic/persistent mastitis (Group D), each group consisted of 400 samples. The MIR modeling approach was similar to that of the healthy and mastitis cattle. From Table 1 and Figure 2, compared with other model, the “DIFF-SVM-274 wavenumbers” model, developed with the spectral preprocessing method of Difference (DIFF), a modeling algorithm of support vector machine (SVM), and 274 wavenumbers, could be effectively used to diagnose mastitis and chronic/persistent mastitis, with AUCs of 0.87 in the training set and 0.85 in the test set.

## 4. Discussion

### 4.1. The Relationship Between Somatic Cell Counts, Differential Somatic Cell Counts, and Milk Composition

Elevated SCC indicates aggravated mastitis severity, which disrupts mammary epithelial cell function and reduces milk yield, fat synthesis, and casein production. Research indicates that for each unit increase in the somatic cell score (SCS), there is a corresponding decrease of 0.43 kg in milk yield, 0.01 kg in milk fat yield, and 0.01 kg in milk protein yield [20]. Research indicated that milk from high-SCC and infected quarters exhibits significant differences in milk composition compared to milk from low-SCC and normal quarters [31,32]. Studies by Stocco et al. (2020) demonstrated that as the differential somatic cell count (DSCC) increases, the quantity of milk fat decreases, and the milk protein reaches its lowest levels at the highest DSCC values, whereas lactose has a positive correlation with DSCC [33]. Research by Bisutti et al. (2022) shows that both SCC and DSCC influence the content of various proteins in milk [34]. Pegolo et al. (2021) found that SCC and DSCC were associated with changes in milk composition, with SCS negatively correlated with milk quality, and increases in DSCC associated with higher milk yield, casein index, and lactose levels, but lower milk fat percentage and electrical conductivity [35].

While adopting Schwartz’s classification method (Group A: healthy, Groups B: suspicious mastitis, C: mastitis, D: chronic/persistent mastitis) [10] provides a standardized framework for mastitis status, this categorization is not absolute. Potential misclassification may arise from dynamic interactions among causative pathogen, severity of mastitis, stage of lactation, and host responsiveness to intramammary infection [36,37,38]. Despite these limitations, Schwartz’s thresholds (SCC: 200$\;\times \;10^{3}$ cells/mL; DSCC: 65%) remain valuable for herd-level screening [10]. In this study, significant differences (*p* < 0.01) were noted between healthy cattle and those with mastitis and chronic/persistent mastitis. Additionally, significant or highly significant differences (*p* < 0.05, *p* < 0.01) were noted between healthy cattle and suspicious mastitis, and between suspicious mastitis, mastitis, and chronic/persistent mastitis, in terms of milk fat, lactose, total solids, and fat-to-protein ratios. The variations in milk composition among different SCC and DSCC groups suggest differences in the mid-infrared spectra of milk, indicating the potential for developing diagnostic MIR models for different mastitis statuses.

### 4.2. MIR Diagnostic Models for Mastitis with the Criterion of Mastitis Identification of SCC and DSCC

In the management of cattle mastitis, the somatic cell count (SCC) was typically used as a dependent variable, either alone as an indicator of mastitis or in combination with other variables to predict the presence of mastitis in dairy cattle. In 2019, Schwarz et al. collected milk samples from 582 cattle at 42 days and 5 days before drying off to develop diagnostic models for mastitis pathogens using both SCC and DSCC. Their results indicated that the model combining DSCC and SCC for detecting primary pathogens had an AUC of 0.64, while the models using SCC or DSCC alone both had AUCs of 0.62 [39]. In 2020, Dorota Anglart et al. developed a machine learning model for SCC based on days in milk and milking machines’ conductivity, milk yield, average and peak milk flow rates, and milking duration on a German dairy farm, achieving root mean square error (RMSE) ranging from 0.09 to 0.20 [40].

MIR analyzes the energy absorption of chemical bonds or functional groups of organic materials within the mid-infrared spectrum to qualitatively and quantitatively assess the composition of cattle milk. Changes in milk composition and MIR occur when cattle are infected with pathogens [3], and these changes correlate with cattle diseases [41]. SCC and DSCC are crucial indicators for assessing udder health, milk quality, and mastitis [5,7].

The dichotomous models developed in this study displayed varying levels of effectiveness, with the best MIR models being (1) “DIFF-RF-1060 wavenumbers”, which distinguished between healthy cattle and those with mastitis and achieved AUC scores of 1.00 in the training set and 0.80 in the test set, and (2) “DIFF-SVM-274 wavenumbers” for diagnosing mastitis and chronic/persistent mastitis, which achieved AUC scores of 0.87 in the training set and 0.85 in the test set. The establishment of these optimal models holds significant potential for expanding the application scope of SCC and DSCC, reducing testing costs, and enhancing the efficiency of mastitis detection. The MIR model for distinguishing between healthy and suspicious mastitis cattle showed poor performance in the test set and still needs further study, such as incorporating more representative data and exploring different model algorithms. In this study, only MIR data were utilized for modeling to enhance model applicability across diverse farming systems. Future studies could incorporate lactation stage data to refine prediction accuracy.

## 5. Conclusions

This study developed two effective diagnostic models using mid-infrared spectroscopy (MIR) that can be used to diagnose healthy and mastitis cattle, as well as mastitis and chronic/persistent mastitis cattle. The two MIR models demonstrated high diagnostic capabilities for the experimental data; however, further research is needed to improve the efficiency of the models.

## Figures and Tables

**Table 1:** The metrics for mid-infrared spectroscopy (MIR) diagnostic models of udder health status.

Groups	Modeling Algorithm (Spectral Preprocessing Methods)	Modeling Spectral Wavenumbers	Datasets	Model Evaluation Metrics						
				ACCU	SENS	SPEC	PPV	NPV	MCC	AUC
Healthy (Group A) vs. mastitis (Group BCD)										
	KNN (SG)	1060	Train	0.63	0.65	0.61	0.62	0.63	0.26	0.67
			Test	0.59	0.68	0.50	0.57	0.61	0.18	0.64
	NBM (SG)	1060	Train	0.69	0.60	0.78	0.73	0.66	0.38	0.73
			Test	0.62	0.53	0.71	0.64	0.60	0.24	0.69
	RF (DIFF)	1060	Train	1.00	1.00	1.00	1.00	1.00	1.00	1.00
			Test	0.73	0.66	0.81	0.77	0.70	0.47	0.80
	SVM (None)	1060	Train	0.77	0.69	0.85	0.82	0.73	0.55	0.86
			Test	0.73	0.67	0.78	0.75	0.70	0.45	0.79
	LR (None)	1060	Train	0.79	0.75	0.83	0.81	0.77	0.58	0.87
			Test	0.75	0.69	0.80	0.78	0.72	0.49	0.80
	Adaboost (SG)	1060	Train	0.98	0.97	0.98	0.98	0.97	0.95	1.00
			Test	0.70	0.66	0.73	0.71	0.68	0.39	0.79
Healthy (Group A) vs. suspicious mastitis (Group B)										
	Adaboost (SG)	1060	Train	0.99	0.98	0.99	0.99	0.98	0.97	1.00
			Test	0.62	0.56	0.68	0.63	0.61	0.24	0.63
	SVM (DIFF)	212	Train	0.68	0.69	0.67	0.67	0.68	0.35	0.75
			Test	0.58	0.56	0.59	0.58	0.57	0.15	0.63
	SVM (DIFF)	274	Train	0.67	0.68	0.67	0.67	0.67	0.35	0.75
			Test	0.57	0.55	0.59	0.57	0.57	0.14	0.64
Mastitis (Group C) vs. chronic/persistent mastitis (Group D)										
	RF (SG)	1060	Train	1.00	1.00	1.00	1.00	1.00	1.00	1.00
			Test	0.74	0.74	0.75	0.75	0.74	0.49	0.82
	SVM (DIFF)	274	Train	0.80	0.78	0.83	0.82	0.79	0.60	0.87
			Test	0.79	0.73	0.85	0.83	0.76	0.58	0.85

Note: Modeling algorithms: KNN represents K-nearest neighbor, NBM represents Naive Bayes model, RF represents Random Forest, SVM represents support vector machine, LR represents linear regression, Adaboost represents adaptive boosting. Spectral preprocessing methods: SG represents Savitzky–Golay convolution smoothing, DIFF represents Difference, None represents no pre-processing for MIR data. Model evaluation metrics: ACCU represents accuracy, SENS represents sensitivity, SPEC represents specificity, PPV represents positive predictive value, NPV represents negative predictive value, MCC represents Matthews correlation coefficient, AUC represents area under receiver operating characteristic curve. Group A (healthy: 0 ≤ SCC ≤ 200$\;\times \;10^{3}$ cells/mL and DSCC ≤ 65%), Group B (suspicious mastitis: SCC ≤ 200$\;\times \;10^{3}$ cells/mL and DSCC > 65%), Group C (mastitis: SCC > 200$\;\times \;10^{3}$ cells/mL and DSCC > 65%), and Group D (chronic/persistent mastitis: SCC > 200$\;\times \;10^{3}$ cells/mL and DSCC ≤ 65%). Only MIR data were utilized for modeling, with lactation stage excluded.

## Data Availability

The data presented in this study are available upon request from the corresponding author due to data ownership reasons.

## Abbreviations

The following abbreviations are used in this manuscript:

SCC	Somatic cell count
DSCC	Differential somatic cell count
MIR	Mid-infrared spectroscopy
RF	Random forest
KNN	K-nearest neighbor
LR	Linear regression
NBM	Naive Bayes model
Adaboost	Adaptive boosting
SVM	Support vector machine
SG	Savitzky–Golay convolution smoothing
DIFF	Difference
None	No preprocessing for MIR data
ACCU	Accuracy
SENS	Sensitivity
SPEC	Specificity
PPV	Positive predictive value
NPV	Negative predictive value
MCC	Matthews correlation coefficient
AUC	Area under receiver operating characteristic curve
